# Rapid Assessment of Physical Activity and its Association Among Patients with Low Back Pain

**DOI:** 10.7759/cureus.6373

**Published:** 2019-12-13

**Authors:** Syed Muhammad Azfar, Manal Abdulaziz Murad, Syeda Azim, Mukhtiar Baig

**Affiliations:** 1 Orthopedics, Liaquat College of Medicine and Dentistry, Karachi, PAK; 2 Family Medicine, King Abdulaziz University, Jeddah, SAU; 3 Medical Education and Simulation, Dow University of Health Sciences, Karachi, PAK; 4 Medical Education and Simulation, King Abdulaziz University, Jeddah, SAU

**Keywords:** back pain, physical actvity, rapid assessment of physical activity

## Abstract

Objective

To evaluate the physical activity levels in individuals with low back pain (LBP) and its relationship with various demographic variables like age, gender, number of days off from work and nature of occupation.

Methodology

This was a cross-sectional study that was done in a private hospital in Jeddah, Saudi Arabia. Data was collected between the periods of 2017-2018. All patients who attended the orthopedic outpatient department (OPD) with LBP were included in this study and were requested to fill the Rapid Assessment of Physical Activity (RAPA) questionnaire. The gathered data were analyzed through the Statistical Package for Social Sciences (SPSS Inc., Chicago, IL), version 23. One-way analysis of variance (ANOVA) was used to compare the mean difference in RAPA scores between genders, age, occupation and number of leaves from their work.

Results

Three hundred sixty patients visited the orthopedic OPD with the primary complaint of LBP, 318 (88.3%) were male whereas 42 (11.7%) were female. The study revealed that among these patients, 117 (32.5%) led sedentary while 176 (48.9%) led an underactive lifestyle. Furthermore, 246 (68.3%) patients did not do any activity that increases their muscle strength and flexibility.

Conclusion

The findings of this study revealed that low physical activity is prevalent in patients with LBP. Therefore, the importance of physical activity should be highlighted in order to prevent chronic diseases like LBP.

## Introduction

Low back pain (LBP) is a worldwide problem, contributing to the highest number of years lived with disability among all musculoskeletal conditions. LBP has a large ﬁnancial impact, signiﬁcantly burdening economies throughout the world [1]. A study done in Saudi Arabia mentioned that backpain as a topmost diagnosis among 23,000 patients [2]. There are numerous established risk factors for LBP such as heavy physical workload, heavy weightlifting, extreme sports activity, bending and twisting. But on the other hand, lack of physical activity is also considered a risk factor for the development of chronic LBP [3]. Hence, physical activity and exercises that increase flexibility and muscular strength of the lumbar extensor muscles, is important for the prevention of LBP [4]. Too much or too little physical activity might be related to LBP which suggests a relationship between physical activity and LBP is complex [4]. LBP follows a U-shaped curve i.e. too little or too much activity is equally hazardous for spinal health and can cause lower back pain [5]. Physical activity is defined as “any bodily movements produced by skeletal muscles that result in energy expenditure” [6].

It is recognized that physical activity has many benefits in relation to physiological and psychological. It is recommended that an adult should have at least 30-60 min of daily physical activity to earn positive health benefits [7]. It is well known that physical activity can reduce the risk factors of a wide variety of chronic diseases such as cardiovascular and respiratory diseases, diabetes, obesity, and musculoskeletal diseases, but there are conflicting reports about its associations with LBP [7]. 

Low physical activity, sedentary lifestyle, and back pain are related to each other and follow a cycle i.e. prolonged inactivity can increase back pain because the back becomes stiff and weak. And, when pain increases, patients reduce their physical activity and exercise levels, which results in even more back pain and the cycle continues [8]. Being physically inactive is an endemic challenge worldwide and contributes to increasing the burden of several chronic health conditions including back pain [9]; it is necessary to explore the relationship between physical activity level and LBP. By understanding the link between physical activity and LBP, we can better manage and prevent LBP. Thus, the objective of this study is to evaluate the physical activity levels in individuals with LBP and their relationship with various demographic variables i.e. age, gender, and nature of the occupation.

## Materials and methods

This cross-sectional study was done in a secondary care hospital of Jeddah, Saudi Arabia. Data was collected between the periods of 2017-2018. The approval for this study was obtained from the hospital ethical committee. We have used a nine-item, self-administered questionnaire to measure the physical activity of patients with LBP [10]. The original questionnaire was in the English language but it was translated into the Arabic language. This questionnaire has two parts. Rapid Assessment of Physical Activity 1 (RAPA 1) evaluates a wide range of physical activity levels, from sedentary to vigorous activity, while RAPA 2 evaluates the strength and flexibility training. Each question has a ‘Yes’ or ‘No’ option. This questionnaire has adequate convergent validity, good criterion validity [10]. Though this questionnaire is particularly designed to measure the activity level of the older adult, we have used this for all adult age groups.

All patients who presented to the orthopedic outpatient department (OPD) with complaints of LBP were included in this study and they were requested to fill the RAPA 1 and RAPA 2 questionnaire after taking their consent. Demographic variables such as age, gender, level of education, and number of leaves from work were also noted.

The gathered data were analyzed by the Statistical Package for Social Sciences (SPSS Inc., Chicago, IL), version 23. Descriptive-analytic methods were employed to analyze the gathered data. Frequencies and percentages were calculated for qualitative variables and mean ± standard deviation was calculated for quantitative variables. One-way analysis of variance (ANOVA) was used to compare the mean difference in depression, anxiety and stress scores between genders, age, and number of leaves from their work. The p-value less than 0.05 considered statistically significant

## Results

Out of 362 patients who visited the orthopedic OPD with the complaint of LBP, there were 42 (11.7%) females and 318 (88.3%) males; the mean age was 39.82±11.42. The basic characteristics of the study subjects are shown in Table 1.

**Table 1:** Basic characteristics of patients

	N(%) Mean±SD
Gender	
Female	42(11.7)
Male	318(88.3)
Marital status	
Forced single	207(57.5)
Married	123(34.2)
Single	30(8.3)
Age	39.82±11.42
<=30	76(21.1)
31-40	139(38.6)
41-50	74(20.6)
>50	71(19.7)
Education	
College Level	109(30.3)
High school Level	101(28.1)
Primary school Level	150(41.7)
Off work due to severity	
Off- work <4 days	141(39.2)
Off- work >4 days	18(5)
No off work	144(40)
No disability	53(14.7)
No pain	4(1.1)
Weight (Kg)	78.43±13.60
Height (cm)	166.06±10.83
BMI (Kg/m2)	28.24±4.61
Underweight	0(00)
Normal	36(10)
Overweight	42(11.7)
Obese	282(78.3)

Table 2 shows the distribution of patients according to RAPA 1 and RAPA 2. According to RAPA 1, most of the study subjects were sedentary 117 (32.5%) and underactive 176 (48.9%). According to RAPA 2, the majority of the study subjects had no activity to improve muscle strength and flexibility 246 (68.3%) and activity to improve strength 90 (25%).

**Table 2:** Distribution of patients according to Rapid Assessment of Physical Activity (RAPA 1 and RAPA 2)

	N(%)
RAPA1	
Sedentary	117(32.5)
Underactive	176(48.9)
Underactive regular light activities	50(13.9)
Underactive regular	6(1.7)
Active	11(3.1)
RAPA2	
No Activity to improve muscle strength or flexibility	246(68.3)
Activity to improve strength	90(25)
Activity to improve flexibility	17(4.7)
Both (strength and flexibility activity)	7(1.9)

The RAPA 1 score is significantly correlated with gender (<.001), and the nature of jobs (<.001) while no correlation was found with age, body mass index (BMI), and off work due to the severity of pain among patients with LBP (Table 3). The males were more sedentary and underactive while having LBP. The manual workers were more sedentary and 'underactive regular light activities' while non-manual was sedentary and underactive while having LBP. People working on desk jobs were more sedentary and underactive while having LBP and the majority of housewives were sedentary while having LBP.

The RAPA 2 score is significantly correlated with gender (<.01), age (<.01), and nature of jobs (<.001), and while no correlation was found with BMI, and off work due to severity of pain among patients with LBP (Table 4). Most females had non-activity while having LBP and non-activity was also common among the age group >50 years, 31-40 years, and 41-50 years. The housewives and non-manual workers and desktop workers had more non-activity while manual workers had more activity for strength while having LBP. 

**Table 3:** Correlation of Rapid Assessment of Physical Activity (RAPA 1) with age, gender, body mass index, off work due to the severity of pain, and nature of job among patients with low back pain

Variable	RAPA 1					P-value
	Sedentary	Underactive	Underactive regular light activities	Underactive regular	Active	
Gender						
Female N=42	31(73.8%)	9(21.4%)	2(4.8%)	0(0.0%)	0(0.0%)	.001
Male N=318	86(27.0%)	167(52.5%)	48(15.1%)	6(1.9%)	11 (3.5%)	
Age						
<=30 N=76	15(19.7%)	41(53.9%)	16(21.1%)	1(1.3%)	3(3.9%)	.14
31-40 N=139	41(29.5%)	73(52.5%)	19(13.7%)	2(1.4%)	4(2.9%)	
41-50 N=74	27(36.5%)	35(47.3%)	8(10.8%)	2(2.7%)	2(2.7%)	
>50 N=71	34(47.9%)	27(38.0%)	7(9.9%)	1(1.4%)	2(2.8%)	
BMI						
Normal N=36	8(22.2%)	20(55.6%)	5(13.9%)	0(0.0%)	3(8.3%)	.43
Overweight N=42	14(33.3%)	19(45.2%)	8(19.0%)	0(0.0%)	1(2.4%)	
Obese N=282	95(33.7%)	137(48.6%)	37(13.1%)	6(2.1%)	7(2.5%)	
Off work because of severity						
Absent <4 days N=141	38(27.0%)	74(52.5%)	24(17.0%)	1(0.7%)	4(2.8%)	.69
Absent >4 days N=18	4(22.2%)	10(55.6%)	3(16.7%)	0(0.0%)	1(5.6%)	
No absence N=144	51(35.4%)	66(45.8%)	19(13.2%)	4(2.8%)	4(2.8%)	
No disability N=53	21(39.6%)	25(47.2%)	4(7.5%)	1(1.9%)	2(3.8%)	
No pain N=4	3(75.0%)	1(25.0%)	0(0.0%)	0(0.0%)	0(0.0%)	
Job nature						
Manual N=118	10(8.5%)	55(46.6%)	39(33.1%)	5(4.2%)	9(7.6%)	.001
Non manual 147	45(30.6%)	94(63.9%)	6(4.1%)	1(0.7%)	1(0.7%)	
Desk N=53	26(49.1%)	22(41.5%)	4(7.5%)	0(0.0%)	1(1.9%)	
Housewife N=42	36(85.7%)	5(11.9%)	1(2.4%)	0(0.0%)	0(0.0%)	

**Table 4:** Correlation of Rapid Assessment of Physical Activity (RAPA 2) with age, gender, body mass index, off work due to the severity of pain, and nature of job among patients with low back pain

Variable	RAPA 2				P-value
	No activity	Activity for strength	Activity for flexibility	Both	
Gender					
Female	38(90.5%)	3(7.1%)	1(2.4%)	0(0.0%)	0.01
Male	208 (65.4%)	87(27.4%)	16(5.0%)	7(2.2%)	
Age					
<=30	41(53.9%)	27(35.5%)	4 (5.3%)	4(5.3%)	0.01
31-40	98(70.5%)	33(23.7%)	8(5.8%)	0(0.0%)	
41-50	51(68.9%)	18(24.3%)	2(2.7%)	3(4.1%)	
>50	56(78.9%)	12(16.9%)	3(4.2%)	0(0.0%)	
BMI					
Normal	24(66.7%)	9(25.0%)	2(5.6%)	1(2.8%)	.98
Overweight	29(69.0%)	11(26.2%)	2(4.8%)	0(0.0%)	
Obese	193(68.4%)	70(24.8%)	13(4.6%)	6(2.1%)	
Off work because of severity					
Absent <4 days	88(62.4%)	44(31.2%)	6(4.3%)	3(2.1%)	.50
Absent >4 days	9(50.0%)	7(38.9%)	1(5.6%)	1(5.6%)	
No absence	108(75.0%)	26(18.1%)	8(5.6%)	2(1.4%)	
No disability	38(71.7%)	12(22.6%)	2(3.8%)	1(1.9%)	
No pain	3(75.0%)	1(25.0%)	0(0.0%)	0(0.0%)	
Job nature					
Manual	41(34.7%)	61(51.7%)	12(10.2%)	4(3.4%)	.001
Non manual	123(83.7%)	21(14.3%)	2(1.4%)	1(0.7%)	
Desk	43(81.1%)	6(11.3%)	2(3.8%)	2(3.8%)	
Housewife	39(92.9%)	2(4.8%)	1(2.4%)	0(0.0%)	

Linear regression analysis shows that RAPA 1 was negatively associated with age (=.005), and job nature (<.001) (Table 5). Linear regression analysis indicates that RAPA 2 was negatively associated with age (P=.005), and job nature (<.001) (Table 6). 

**Table 5:** Linear regression showing association of RAPA 1 with age, gender, body mass index, off work due to severity of pain, and nature of job among patients with low back pain RAPA: Rapid Assessment of Physical Activity.

Model	Beta Coefficients	t statistics	p-value	95% Confidence Interval for Beta	
				Lower Bound	Upper Bound
(Constant)	3.495	7.587	.000	2.589	4.401
Age	-.010	-2.814	.005	-.018	-.003
Gender	-.029	-.192	.847	-.329	.270
BMI	-.008	-.879	.380	-.026	.010
Off work because of severity	.037	1.003	.316	-.035	.109
Job Nature	-.463	-9.034	.000	-.564	-.362

**Table 6:** Linear regression showing association of RAPA 2 with age, gender, body mass index, off work due to severity of pain, and nature of job among patients with low back pain RAPA: Rapid Assessment of Physical Activity.

Model	Beta Coefficients	t statistics	p-value	95% Confidence Interval for Beta	
				Lower Bound	Upper Bound
(Constant)	2.575	6.887	.000	1.840	3.310
Age	-.008	-2.788	.006	-.014	-.002
Gender	-.096	-.773	.440	-.339	.148
BMI	-.005	-.704	.482	-.020	.009
Off work because of severity	-.003	-.115	.908	-.062	.055
Job Nature	-.247	-5.938	.000	-.329	-.165

## Discussion

In this study, we found that patients with LBP who are physically inactive are at increased risk for LBP. According to our data RAPA 1, most of the patients with back pain were sedentary 117 (32.5%) and underactive 176 (48.9%). Citko et al. concluded similar results in their study and found that the sedentary lifestyle significantly increased the incidence of recurring LBP [11]. It is evident that a person who sits too much was more likely to experience back pain [12]. In contrast, a study done in Netherland revealed that both extremes of the physical activity pattern are associated with a higher prevalence of chronic low back pain (CLBP) complaints, suggesting that the relation between the level of activity and LBP is complex [5]. That is why it is recommended that patients who are experiencing back pain should not avoid exercise completely because light exercise and movement are the natural stimuli for the healing process [4].

In this study, RAPA scores are significantly correlated with gender. It showed that female patients have less physical activity as compared to male patients. Dumiht et al. also uncovered, in the analysis of multiple studies, that female physical inactivity was higher than the male, and which is the significant risk factor of several medical conditions including LBP [13]. The female patients in our study are less active physically as compared to the male patients, perhaps because most of the female patients visiting the clinic were housewives and generally have a sedentary lifestyle. According to the World Health Organization (WHO), there are numerous elements that limit women's physical activity e.g. they have care-giving roles for other family members which limits their physical activities [14].

The nature of jobs is another risk factor found to be significantly associated with physical inactivity and backpain. The manual workers were less sedentary and underactive as compared to non-manual patients while having LBP. Most of the non-manual patients working on desk jobs were sedentary and underactive while having LB. Several other studies also show that there is a considerable association between the nature of jobs and LBP [1,3-4]. It is assumed that lack of physical activity, resulting from the sedentary lifestyle, results in the reduction of muscular power and strength which leads to a reduced ability of the vertebral disc to maintain a normal concentration of water [15]. Our studies demonstrated that the sedentary lifestyle was led by nearly half (49.59%) of all subjects and as many as 67.59% in the group with recurrent LBP. These findings suggest that patients who have a sedentary lifestyle are exposed to the risk of back pain. So, we as a physician should educate our patients to reduce sitting time in the workplace and at home and try to incorporate feasible physical activity interventions to reduce the risk of back pain [16].

Our study reveals that about 68% of patients with LBP do not do any activity to increase their muscle strength and flexibility. This finding is similar to other research done on the adolescent population which revealed that muscle strength and flexibility exercise reduced the risk of LBP in later life [16]. Reduced muscle strength and poor body flexibility are the established risk factors for developing LBP but a study by Sandler et al. (2014) showed no association between muscle strength and LBP. They concluded that there is evidence that the treatment of LBP with flexibility exercises can provide relief of symptoms; however, flexibility does not seem to reduce the risk development of LBP [17].

We also found, in our study, that more than 75% of patients with LBP who are above 50 do not engage in any physical activity for muscle strengthening and physical activity. The sedentary lifestyles often predominate in older age which can cause several health issues including LBP. Physicians and health-promoting authorities have a responsibility to promote physical activity amongst older people [11]. Even though obesity is often correlated with low physical activity in LBP patients, surprisingly we could not find any relation between these variables [18].

Limitations

This is the cross-sectional study and only provides a snapshot of the problem. Furthermore, a perception-based questionnaire might not represent the actual reality. The questionnaire used in this study (RAPA) is mainly used for the old adult population, but we used in all adult age group. Therefore, the reliability of this scale required further explored in other age groups.

## Conclusions

In conclusion, the findings of this study revealed that LBP and low physical activity are related to each other. A notable number of patients with LBP led a sedentary lifestyle. Therefore, these findings acknowledge the importance of physical activity in the prevention of chronic diseases like LBP. Clinicians should encourage their patients to involve in some sort of physical activity to prevent LBP.
